# CropCLR-Wheat: A Label-Efficient Contrastive Learning Architecture for Lightweight Wheat Pest Detection

**DOI:** 10.3390/insects16111096

**Published:** 2025-10-25

**Authors:** Yan Wang, Chengze Li, Chenlu Jiang, Mingyu Liu, Shengzhe Xu, Binghua Yang, Min Dong

**Affiliations:** 1 China Agricultural University, Beijing 100083, China; 2 Artificial Intelligence Research Institute, Tsinghua University, Beijing 100084, China

**Keywords:** wheat pest recognition, agricultural AI deployment, contrastive learning, lightweight fine-tuning, few-shot learning

## Abstract

To address prevalent challenges in field-based wheat pest recognition—namely, viewpoint perturbations, sample scarcity, and heterogeneous data distributions—a pest identification framework named CropCLR-Wheat is proposed, which integrates self-supervised contrastive learning with an attention-enhanced mechanism. By incorporating a viewpoint-invariant feature encoder and a diffusion-based feature filtering module, the model significantly enhances pest damage localization and feature consistency, enabling high-accuracy recognition under limited-sample conditions. In 5-shot classification tasks, CropCLR-Wheat achieves a precision of 89.4%, a recall of 87.1%, and an accuracy of 88.2%; these metrics further improve to 92.3%, 90.5%, and 91.2%, respectively, under the 10-shot setting. In the semantic segmentation of wheat pest damage regions, the model attains a mean intersection over union (mIoU) of 82.7%, with precision and recall reaching 85.2% and 82.4%, respectively, markedly outperforming advanced models such as SegFormer and Mask R-CNN. In robustness evaluation under viewpoint disturbances, a prediction consistency rate of 88.7%, a confidence variation of only 7.8%, and a prediction consistency score (PCS) of 0.914 are recorded, indicating strong stability and adaptability. Deployment results further demonstrate the framework’s practical viability: on the Jetson Nano device, an inference latency of 84 ms, a frame rate of 11.9 FPS, and an accuracy of 88.2% are achieved. These results confirm the efficiency of the proposed approach in edge computing environments. By balancing generalization performance with deployability, the proposed method provides robust support for intelligent agricultural terminal systems and holds substantial potential for wide-scale application.

## Simple Summary

To tackle challenges in field-based wheat pest recognition—such as viewpoint variations, limited samples, and heterogeneous data—this study proposes CropCLR-Wheat, a self-supervised contrastive learning framework enhanced with attention mechanisms. The model employs a viewpoint-invariant encoder and a diffusion-based feature filter to improve feature consistency and pest damage localization. In few-shot settings, it achieves accuracies of 88.2% (5-shot) and 91.2% (10-shot), and a segmentation mIoU of 82.7%, surpassing SegFormer and Mask R-CNN. Robustness tests show a prediction consistency of 88.7% and a PCS of 0.914, confirming stability under viewpoint perturbations. Deployed on a Jetson Nano, the system reaches 84 ms inference latency and 11.9 FPS, demonstrating strong efficiency for edge-based intelligent agriculture applications.

## 1. Introduction

In the context of continuous global population growth and escalating pressure on agricultural resources, ensuring food security has emerged as a critical strategic imperative [1,2]. As one of the three major staple crops worldwide, wheat plays an essential role in maintaining the stability of global food supply through its steady and abundant yields. However, yield losses caused by complex biotic factors during the growth cycle of wheat—particularly infestations of major insect pests such as *Sitodiplosis mosellana*, *Mythimna separata*, and *Tetranychus cinnbarinus*—represent one of the principal challenges threatening this stability [3,4]. From an entomological and crop physiological perspective, these pests exhibit distinct behavioral and ecological characteristics that intensify their impact on wheat growth. *Sitodiplosis mosellana* larvae feed within wheat florets, directly impairing kernel development and causing substantial yield reductions [5]; *Mythimna separata* (the oriental armyworm) displays migratory outbreaks that can rapidly destroy entire leaf canopies [6]; and *Tetranychus cinnbarinus* (the carmine spider mite) causes chronic damage by puncturing epidermal tissues and disrupting photosynthetic efficiency [7]. The physiological stress induced by such infestations leads to reduced chlorophyll content, impaired transpiration regulation, and weakened resistance to secondary diseases. Moreover, the dynamic interactions between pest population development and crop growth stage—mediated by temperature, humidity, and canopy microclimate—introduce significant spatiotemporal variability, complicating both manual monitoring and model-based prediction [8,9]. Consequently, the precise and timely identification and early warning of these pest infestations are of immeasurable value for enhancing wheat productivity, guiding targeted irrigation and pesticide application, and reducing the overuse of chemical pesticides [9]. In recent years, artificial intelligence technologies represented by deep learning have achieved breakthroughs in image recognition, offering new prospects for the automated diagnosis of wheat growth conditions [8,10]. Nonetheless, when these models—which demonstrate excellent performance under laboratory conditions—are deployed in the complex and variable environments of actual wheat fields, a performance degradation is often observed, thereby exposing challenges in practical applications.

Currently, most crop growth recognition techniques are predominantly based on the supervised learning paradigm [11,12]. To pursue higher recognition accuracy, researchers have focused on developing deeper and more complex network architectures. For instance, Ullah et al. proposed a deep convolutional neural network named DeepPestNet, which employs image rotation and data augmentation strategies to alleviate data scarcity. By incorporating a multi-scale convolutional structure to automatically extract discriminative features, DeepPestNet achieved an impressive recognition accuracy of 98.92% across nine pest categories on the Kaggle pest dataset [13]. However, such methods typically involve complex model structures and substantial computational costs, making them difficult to deploy for lightweight and real-time analysis in field environments [14]. To address deployment challenges, other studies have focused on model lightweighting. Ali et al. proposed a convolutional neural network-based deep model that effectively reduces data volume while improving recognition accuracy. They designed a lightweight framework, Faster-PestNet, which integrates MobileNet into an enhanced Faster R-CNN architecture and achieved a recognition accuracy of 95.24% on the large-scale IP102 dataset [15]. Similarly, Janarthan et al. introduced a lightweight residual attention network, LiRAN, which merges four simplified attention mask modules into MobileNetV2 and leverages an inverted residual structure to enhance feature representation. Despite containing only 2.37M parameters, LiRAN maintained high recognition performance under complex backgrounds and limited samples [16]. These studies have made notable progress in model compression and inference efficiency; nevertheless, their success still heavily depends on large-scale, high-quality, and manually annotated datasets for supervised training, leading to fundamental paradigm constraints in practical wheat production scenarios. First, obtaining high-quality annotations is extremely costly. Unlike standardized pest image datasets, wheat pest and disease symptoms vary substantially—features such as leaf yellowing, curling, or feeding marks differ depending on pest species and developmental stages. As a result, accurate labeling heavily relies on agronomic experts, whose work is often inefficient, subjective, and impractical for large-scale data processing [17]. Second, the limited diversity of annotated data restricts model generalization. The inherent heterogeneity of wheat production—reflected in crop varieties, geographical environments, and growth stages—leads to significant domain shifts [18]. Consequently, models developed for specific fields or pest types often experience severe performance degradation when applied to new varieties or regions, posing major challenges to cross-scenario adaptability [18]. Most critically, existing models generally lack robustness to dynamic environmental changes; their performance declines sharply with variations in illumination conditions or camera viewpoints, which directly constrains their deployment in automated inspection platforms such as UAV-based monitoring systems [19,20]. Together, these challenges point to a central contradiction: the success of supervised learning relies on idealized data, whereas real-world agricultural environments are inherently non-ideal.

**Figure 1:**
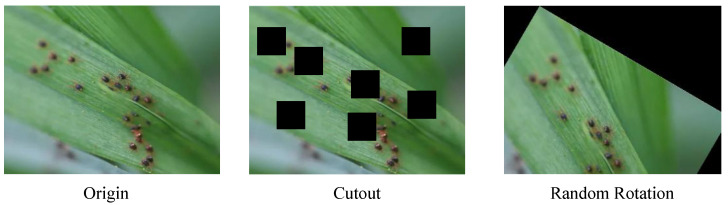
Examples of image augmentation methods that destroy the fragile semantics of agricultural images.

Self-supervised contrastive learning has emerged as a transformative paradigm that challenges conventional supervised learning frameworks [21,22,23,24]. By training models to autonomously distinguish between “similar” and “dissimilar” samples, it enables the extraction of rich semantic representations from unlabeled data. However, when applied directly to agricultural vision tasks, existing frameworks such as SimCLR and MoCo reveal critical limitations [25]. These approaches rely heavily on generic data augmentations to generate positive pairs—an assumption that works well for natural image datasets but fails in the agricultural domain. In wheat field pest imagery, visual semantics are fragile and domain-specific: subtle morphological or textural variations caused by insect feeding often serve as key diagnostic cues, as shown in Figure 1. Standard augmentations designed for natural images can easily distort or erase these delicate patterns, producing pseudo-positive samples that mislead the model and degrade representation quality [26]. Moreover, unlike SimCLR and MoCo, which presume that the “object of interest” is globally salient and well-centered, agricultural scenes contain multiple uncertain learning targets—including pest bodies, damaged leaf textures, and background noise. Without domain-specific guidance, traditional contrastive methods tend to prioritize visually dominant global features, neglecting the fine-grained, pest-induced damage essential for accurate recognition. These challenges indicate that directly transplanting existing contrastive frameworks is insufficient for complex agricultural environments.

**Figure 2:**
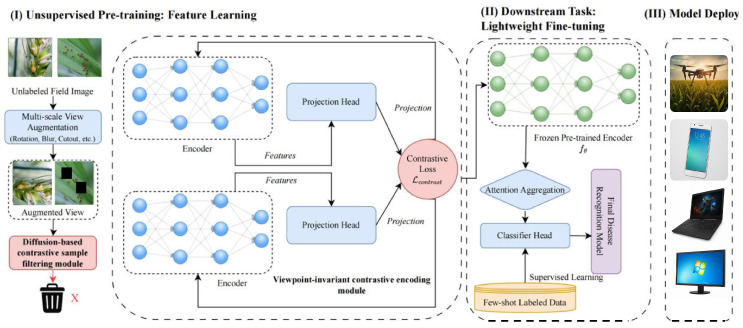
Overview of CropCLR-Wheat framework. A two-stage pipeline integrating unsupervised contrastive pretraining, lightweight fine-tuning with attention, and deployment on edge and mobile platforms for robust wheat pest recognition.

To address these challenges and truly unlock the potential of self-supervised learning in agriculture, a self-supervised pest recognition framework specifically designed for complex field environments—CropCLR-Wheat—is proposed in this study, as shown in Figure 2. CropCLR-Wheat fundamentally resolves the practical difficulties of deploying self-supervised learning in agricultural scenarios through three key innovative designs:

1. **A viewpoint-invariant contrastive encoding module was proposed to handle posture and environmental variations.** To cope with dynamic and diverse shooting conditions in the field, a multi-scale viewpoint augmentation strategy was designed, compelling the model to maintain consistency in pest feature recognition under varying illumination, angles, and distances. This module enhances environmental robustness by enforcing cross-view feature alignment. As demonstrated in the ablation study, introducing this module improved classification accuracy by 4.2%, confirming its effectiveness in mitigating viewpoint perturbations.
2. **A semantic enhancement and sample filtering module was designed to ensure the quality of learning signals.** To address the challenges of “pseudo-positive samples” and “background interference,” a semantic preservation mechanism was introduced based on diffusion consistency filtering. This mechanism intelligently discards augmented samples that distort critical pest-related features, ensuring that the model emphasizes true pest-induced semantics rather than irrelevant contextual noise. Quantitative results show that applying this filtering strategy increased representation purity and boosted downstream classification accuracy by 3.3%, demonstrating its role in refining contrastive learning quality.
3. **A lightweight fine-tuning module based on attention aggregation was introduced to enable efficient transfer.** After unsupervised pretraining, a lightweight attention aggregation module was employed, requiring only a small number of labeled samples to adapt general representations to fine-grained pest categories. This module performs attention head pruning and token-level aggregation, allowing rapid adaptation with reduced computational overhead. Experimental results indicate that, under five-shot fine-tuning, the proposed module outperformed traditional fine-tuning strategies (e.g., ResNet-50) by 5.2% in accuracy, effectively addressing the challenge of limited annotations during early pest outbreak detection.

The proposed CropCLR-Wheat framework was extensively validated on multiple datasets. Experimental results demonstrated that by pretraining on the real-field unlabeled dataset CropField-U, CropCLR-Wheat comprehensively outperformed existing self-supervised methods such as SimCLR and BYOL in few-shot fine-tuning tasks and exhibited outstanding efficiency and stability in field deployment tests. This proves that the proposed framework is not only theoretically innovative but also possesses significant potential for practical application and large-scale adoption. The remainder of this paper is organized as follows: the first section introduces the research background, challenges, and core innovations; the second section details the dataset construction and the design of the CropCLR model; the third section presents and deeply analyzes the experimental results; and the fourth section summarizes the research findings and outlines future directions.

## 2. Materials and Methods

### 2.1. Dataset Construction

#### 2.1.1. Data Collection

**Figure 3:**
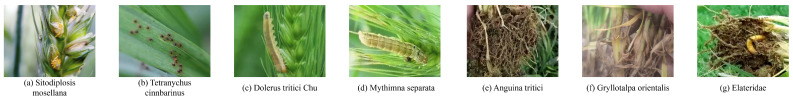
Example images of wheat pest categories, including *Sitodiplosis mosellana* (**a**), *Tetranychus cinnbarinus* (**b**), *Dolerus tritici* Chu (**c**), *Mythimna separata* (**d**), *Anguina tritici* (**e**), *Gryllotalpa orientalis* (**f**), and *Elateridae* (**g**).

To enable the generalized identification of multiple wheat pest categories, an unlabeled image dataset named CropField-U was constructed, covering diverse locations, time periods, and environmental conditions, as shown in Table 1 and Figure 3.

The data collection process spanned nearly one year, from June 2023 to October 2024. The primary collection sites were selected as Yutian County in Tangshan City, Hebei Province, and Wuyuan County in Bayannur City, Inner Mongolia Autonomous Region, in order to encompass ecological diversity within representative wheat-producing areas in northern China. Tangshan represents the Huang-Huai winter wheat zone, characterized by fertile soil and convenient irrigation, which facilitates the observation of field progression for major wheat pests such as *Sitodiplosis mosellana*, *Dolerus tritici* Chu, *Mythimna separata*, and *Tetranychus cinnbarinus*. These species differ markedly in their life cycle timing and infestation behavior: *S. mosellana* undergoes pupation in early spring and causes spikelet damage during heading; *M. separata* exhibits migratory outbreaks in midsummer, defoliating large field areas within days; and *T. cinnbarinus* proliferates under high-temperature and low-humidity conditions in late growth stages, often persisting through multiple overlapping generations. In contrast, Bayannur is located in a cold, arid, and semi-arid region with strong winds and large diurnal temperature variations, making it suitable for capturing representative infestation patterns of soil-dwelling and root-associated pests such as *Anguina tritici*, *Gryllotalpa orientalis*, and *Elateridae*. The unique soil temperature and moisture dynamics in this region influence the overwintering and emergence rhythms of these subterranean species, offering valuable ecological diversity for dataset construction. Additionally, the distinct phenological calendar between the two regions—early sowing and harvest in Tangshan versus delayed germination and extended growth in Bayannur—allowed for the documentation of pest–crop interactions across different wheat developmental stages. To enrich the diversity and generalization potential of the dataset, several high-quality open-source datasets were integrated, including wheat leaf images from PlantVillage and agricultural environmental images from the AI Challenger platform (Beijing, China), to supplement standard samples with high feature clarity and minimal background noise. Field data were primarily acquired using agricultural drones (DJI Phantom 4 Pro; DJI, Shenzhen, China) and ground-based handheld devices (iPhone 13 Pro and RICOH G900). A multi-angle and variable-zoom strategy was adopted to capture both plant group status and leaf-level details, ensuring the inclusion of macroscopic growth conditions and microscopic pest-induced damage. UAV flights were conducted at altitudes ranging from 5 to 15 m, under natural lighting conditions between 9:00–11:00 AM and 4:00–6:00 PM to avoid overexposure and shadow occlusion caused by intense midday sunlight. Ground photography employed both oblique and top-down perspectives to emphasize close-up features such as feeding traces, leaf chewing patterns, discoloration, and structural damage caused by pest activity. All raw images were saved in JPEG format with a minimum resolution of 3024 × 4032 to meet the spatial resolution requirements of subsequent contrastive learning. Prior to preprocessing, no manual annotation was applied to the images, preserving natural variations in lighting, weed interference, and motion blur, thereby enhancing model robustness and generalization to real-world field perturbations.

#### 2.1.2. Data Preprocessing

To ensure robustness of the model under varying scenarios, temporal conditions, and field interferences, a systematic preprocessing and augmentation strategy was developed based on the CropField-U dataset. This strategy addressed multiple dimensions, including image resolution normalization, color consistency adjustment, blur robustness, and spatial perturbation. During the preprocessing stage, all raw images were resized to a standardized format. Given the substantial resolution differences among images captured by various devices (ranging from 3024×4032 to 3840×2160), all images were rescaled to a square format of 512×512 to meet model input requirements and enhance computational efficiency. Bilinear interpolation was used for resizing. In cases where the original image was non-square, center cropping was first performed to retain the main visual content, followed by scaling and zero-padding to prevent deformation or displacement of target regions. To enhance color consistency under different lighting conditions, histogram equalization was applied to normalize the luminance channel, and automatic white balance adjustment was achieved using the gray world algorithm. This process mitigated lighting discrepancies caused by temporal variations (e.g., dawn, dusk, and cloudy conditions), enabling the model to focus on the intrinsic color and texture patterns associated with pest infestation. A multi-scale perspective augmentation strategy was further designed to construct contrastive learning sample pairs, incorporating spatial and blur-based perturbations. Spatial augmentations included random rotation with $\theta ∼U(-30^{\circ },+30^{\circ })$, random horizontal flipping, and random scaling with a factor s∼U(0.8,1.2), emulating geometric distortions introduced by varying UAV perspectives or ground device angles. Blur augmentation was performed by applying a 3×3 Gaussian kernel, where the standard deviation σ∼U(0.5,1.5), simulating real-world blur due to wind or focal fluctuations.

In addition, cutout-based regional occlusion augmentation was employed to enhance model robustness against occlusion and background interference from weeds. Specifically, one or more rectangular masks were randomly generated within each image, with the area *S* of each mask sampled from S∼U(0.05A,0.2A), where *A* denotes the total image area. These masks were inserted at random locations using gray or zero-value pixels to simulate natural scenarios such as leaf overlap or branch occlusion. During the contrastive learning phase, any image *x* was subjected to two distinct augmentation pipelines $t_{i}\left(\cdot \right)$ and $t_{j}\left(\cdot \right)$, yielding two augmented views $x_{i}=t_{i}\left(x\right)$ and $x_{j}=t_{j}\left(x\right)$ that formed a positive pair $\left(x_{i},x_{j}\right)$, formally defined as follows:

(1) $$x_{i}=t_{i}\left(x\right),\;x_{j}=t_{j}\left(x\right),\;\left(x_{i},x_{j}\right)\in P,$$

where P denotes the set of all positive sample pairs. Contrastive loss was subsequently applied to learn feature similarity within positive pairs while distinguishing negative samples. All aforementioned augmentation operations were implemented using custom pipelines within the ‘torchvision.transforms‘ module in PyTorch 2.5.1, supporting batch-wise parallelization and GPU acceleration. This ensured sufficient perturbation diversity and semantic consistency in the samples generated for each training epoch, thereby enhancing model generalization and practical deployment capability under real-world field conditions.

### 2.2. Proposed Method

**Figure 4:**
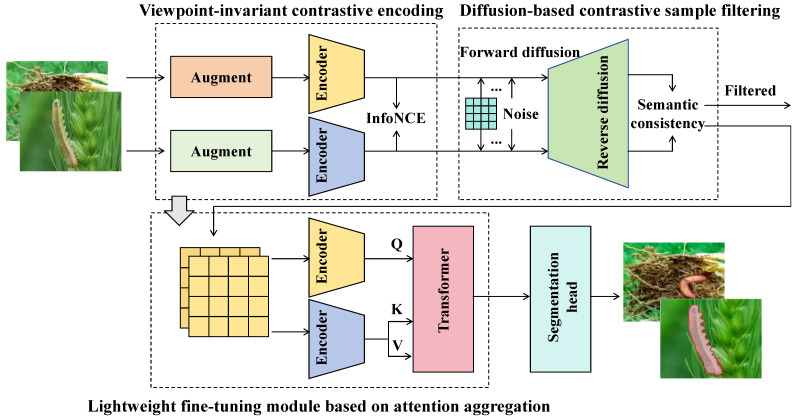
Schematic diagram of the overall architecture of CropCLR-Wheat.

The proposed CropCLR-Wheat framework, as shown in Figure 4, is designed as a modular pipeline centered on self-supervised contrastive learning, aiming to achieve robust recognition of major in-field wheat pests, encompassing diverse infestation patterns caused by seven representative species. After undergoing preprocessing and augmentation, field images are first processed by a viewpoint-invariant contrastive encoding module. Positive sample pairs are generated via two distinct augmentation pipelines and are subsequently mapped into the contrastive learning space through an encoder and projection head. The features are optimized using InfoNCE loss to ensure consistency. These augmented views are then input into a diffusion-based filtering module, where a pixel-level semantic graph is constructed to evaluate structural similarity. Only high-consistency sample pairs are retained for training, effectively mitigating semantic drift. Following self-supervised training, the encoder backbone is frozen, and a small set of labeled samples is introduced for fine-tuning. During this phase, an attention aggregation module is incorporated to enhance critical feature responses. Finally, discriminative features are extracted for accurate pest category prediction. The three-stage structure maintains semantic coherence and structural continuity, balancing adaptive feature learning without labels and high-accuracy, efficient fine-tuning, thereby enabling practical deployment and generalization in agricultural edge scenarios.

#### 2.2.1. Viewpoint-Invariant Contrastive Encoding Module

**Figure 5:**
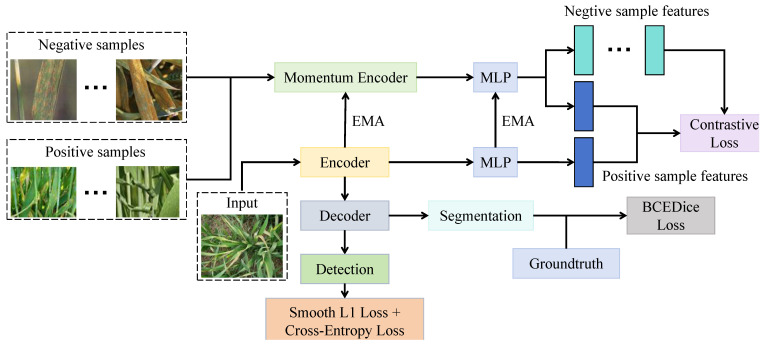
This diagram illustrates the structure of the viewpoint-invariant contrastive encoding module designed to address pose and viewpoint variations in field imagery. The input image is processed by a shared encoder, whose weights are updated via exponential moving average (EMA) to produce a momentum encoder. Positive and negative samples are fed into the momentum encoder and projected via MLP for contrastive alignment. The encoder output is simultaneously decoded to yield segmentation or detection predictions, supervised by a BCEDice loss for segmentation or a combination of Smooth L1 Loss and cross-entropy loss terms for detection.

The viewpoint-invariant contrastive encoding module serves as the foundation of the entire self-supervised framework in CropCLR-Wheat. Its primary objective is to address the substantial appearance variations in field images caused by different viewing angles, illumination conditions, and shooting distances and to construct stable and generalizable feature representations. Inspired by the MoCo architecture, as shown in Figure 5, the module comprises a primary encoder $f_{\theta }$ and a momentum encoder $f_{\theta^{\prime }}$, which share identical structures but are updated independently. The primary encoder extracts semantic features from the current mini-batch of augmented images, while the momentum encoder constructs a stable contrastive queue. Parameters of the momentum encoder are updated using an exponential moving average, following $\theta^{\prime }\leftarrow m\theta^{\prime }+\left(1-m\right)\theta$, where *m* denotes the momentum coefficient (set to 0.999 in this study). In this update rule, the new parameter set $\theta^{\prime }$ of the momentum encoder is computed as a weighted combination of its previous parameters and the current parameters θ of the primary encoder, where *m* controls the update speed. A larger *m* value results in slower updates, allowing the momentum encoder to evolve more smoothly and maintain a stable target representation across training iterations. This design effectively prevents representation collapse and ensures consistency between the two encoders in the contrastive learning process. Each image is processed by two distinct augmentation operations $t_{i}\left(\cdot \right)$ and $t_{j}\left(\cdot \right)$ and then fed into the primary and momentum encoders, respectively, resulting in the generation of positive sample features $z_{i}=g_{\theta }\left(f_{\theta }\left(t_{i}\left(x\right)\right)\right)$ and $z_{j}=g_{\theta^{\prime }}\left(f_{\theta^{\prime }}\left(t_{j}\left(x\right)\right)\right)$. Here, *g* represents an MLP projection head consisting of two fully connected layers (with dimensions 512→128→128), interleaved with a ReLU activation function. The encoder backbone is based on ResNet-50, with the parameters of the first four stages initialized using ImageNet pretrained weights to ensure stable feature extraction. During each forward pass, the positive pair $\left(z_{i},z_{j}\right)$ is compared against all negative samples in the queue using the InfoNCE contrastive loss function:

(2) $$L_{contrast}=-\log\frac{\exp(\mathrm{sim}\left(z_{i},z_{j}\right)/\tau )}{\exp\left(\mathrm{sim}\left(z_{i},z_{j}\right)/\tau \right)+\sum_{k=1}^{K}\exp\left(\mathrm{sim}\left(z_{i},z_{k}^{-}\right)/\tau \right)},$$

where $\mathrm{sim}\left(a,b\right)=\frac{a^{⊤}b}{\left|a||b\right|}$ denotes cosine similarity, $z_{k}^{-}$ represents negative sample features, which are obtained from a dynamic queue of representations encoded by the momentum encoder $f_{\theta^{\prime }}$, and τ is the temperature parameter (set to 0.2). The training process continuously samples different augmented views and constructs a stable feature space, enabling the model to maintain feature invariance under changes in angle, orientation, and lighting, thereby improving its adaptability to diverse field environments. In practical applications, field images often exhibit significant viewpoint drift and structural noise; for instance, the same leaf may appear substantially different when captured in the morning versus at noon or from different perspectives such as top-down or oblique angles. Traditional supervised approaches typically struggle under such perturbations. In contrast, this contrastive encoding module introduces a momentum encoder and a long-queue negative buffer mechanism, which enables the learning of stable discriminative features without the need for manual labels. As evidenced by the experimental results, this module substantially improves fine-tuning accuracy on multi-angle datasets, particularly in low-shot scenarios such as 5-shot and 10-shot settings, outperforming other contrastive learning methods. Within the CropCLR-Wheat framework, this module plays a critical role in feature extraction and representation alignment, providing high-quality semantic embeddings for subsequent filtering and fine-tuning stages.

#### 2.2.2. Diffusion-Based Contrastive Sample Filtering Module

**Figure 6:**
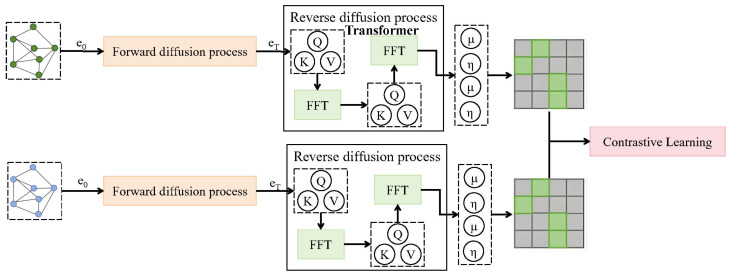
This figure illustrates the architecture of the diffusion-based contrastive sample filtering module, which addresses sample heterogeneity in agricultural pest recognition. The module consists of a forward diffusion process that progressively adds Gaussian noise to the encoded feature map and a reverse reconstruction process implemented via a conditional variational autoencoder with four transformer layers. The reverse process outputs two decoder branches, $\mu_{\theta }\left(e_{t},t\right)$ and $\eta_{\theta }\left(e_{t},t\right)$, representing the mean and variance for semantic reconstruction. The two input feature maps $e_{0}$ correspond to distinct augmented views $t_{i}\left(x\right)$ and $t_{j}\left(x\right)$, which are compared after reconstruction to evaluate semantic consistency and filter out inconsistent samples.

To mitigate the semantic drift introduced by aggressive augmentations in contrastive learning, a diffusion-based contrastive sample filtering module is proposed within the CropCLR-Wheat framework. As shown in Figure 6, the core idea is to reconstruct the original semantic structure in the feature space through a learnable diffusion process. This reconstructed structure is then used to evaluate the semantic consistency of augmented image pairs, thereby filtering out semantically inconsistent negative samples and enhancing the stability and discriminability of training. The module is constructed based on probabilistic diffusion modeling, incorporating graph-based representation learning. It includes both a forward diffusion process and a reverse reconstruction phase and is jointly trained with the contrastive encoding module. Specifically, let the encoded and augmented feature map be denoted by $e_{0}\in R^{H\times W\times C}$, where H=16, W=16, and C=128, indicating the spatial and channel dimensions. The forward diffusion process injects Gaussian noise into $e_{0}$ progressively, forming a sequence ${\left\{e_{t}\right\}}_{t=0}^{T}$ governed by

(3) $$q\left(e_{t}|e_{0}\right)=N\left(e_{t};\sqrt{\alpha_{t}}e_{0},\left(1-\alpha_{t}\right)I\right),$$

where $\alpha_{t}$ denotes the diffusion coefficient at timestep *t*, controlling the noise magnitude. The terminal noisy state $e_{T}$ is treated as a semantically blurred representation and passed to the reverse reconstruction module for semantic recovery. This reverse process is implemented via a conditional variational autoencoder augmented with transformer blocks. The network consists of four transformer layers, each comprising an 8-head multi-head attention mechanism and a feed-forward network (FFN) with hidden dimension 512. Two decoder branches, $\mu_{\theta }\left(e_{t},t\right)$ and $\eta_{\theta }\left(e_{t},t\right)$, are used to approximate the target distribution:

(4) $$p_{\theta }\left(e_{t-1}|e_{t}\right)=N\left(e_{t-1};\mu_{\theta }\left(e_{t},t\right),\eta_{\theta }{\left(e_{t},t\right)}^{2}I\right).$$

The model is optimized using the following variational lower-bound loss:

(5) $$L_{diff}=E_{q}\left[\sum_{t=1}^{T}{\left|e_{t-1}-\mu_{\theta }\left(e_{t},t\right)\right|}^{2}\right].$$

After semantic reconstruction, the resulting representations ${\overset{˜}{e}}_{1}$ and ${\overset{˜}{e}}_{2}$ of the two augmented views are compared. If their structural distance $D_{struct}\left({\overset{˜}{e}}_{1},{\overset{˜}{e}}_{2}\right)$ falls below a predefined threshold δ, the pair is retained as a valid positive sample; otherwise, it is discarded. The structural similarity is measured via a covariance-based metric:

(6) $$D_{struct}\left({\overset{˜}{e}}_{1},{\overset{˜}{e}}_{2}\right)=1-\frac{\mathrm{Tr}(\Sigma_{{\overset{˜}{e}}_{1}}\Sigma_{{\overset{˜}{e}}_{2}})}{{\left|\Sigma_{{\overset{˜}{e}}_{1}}\right|}_{F}\cdot {\left|\Sigma_{{\overset{˜}{e}}_{2}}\right|}_{F}},$$

where $\Sigma_{\overset{˜}{e}}$ denotes the covariance matrix of the feature map, Tr represents the trace operation, and ${\left|\cdot \right|}_{F}$ denotes the Frobenius norm. This diffusion module is jointly trained with the viewpoint-invariant contrastive encoder by sharing the input features $e_{0}$. While the encoder provides contrastive supervision, the diffusion module serves as a semantic consistency filter. The overall joint objective function is defined as follows:

(7) $$L_{joint}=L_{contrast}+\lambda \cdot L_{diff},$$

where λ is a balancing coefficient controlling the strength of the semantic consistency constraint, set to 0.5 in the experiments. This design introduces a continuous semantic constraint via the diffusion process, significantly improving the model’s ability to assess semantic structure consistency under sample heterogeneity. It alleviates the semantic shift issues commonly associated with naive augmentation-based positive pair construction, especially in complex field scenarios characterized by co-occurring pest categories, subtle infestation symptoms, and intricate structures. Moreover, the diffusion module offers strong interpretability and modular decoupling, thereby providing a semantic alignment interface for future multimodal fusion involving external knowledge graphs or environmental sensor data. This capability is expected to further expand the applicability of the CropCLR-Wheat framework.

#### 2.2.3. Lightweight Fine-Tuning Module Based on Attention Aggregation

In the third stage of the CropCLR-Wheat framework, a lightweight fine-tuning module based on an attention aggregation mechanism is proposed to address the learning bottleneck caused by the scarcity of labeled samples in wheat pest recognition scenarios. Inspired by multi-label self-attention token mechanisms, the module simplifies and customizes the traditional transformer architecture to more efficiently focus on critical lesion regions. Unlike standard self-attention mechanisms that model all patch-to-patch relationships, this design introduces a set of learnable class-specific tokens (classification tokens) that attend to image embeddings, thereby capturing class-relevant local response patterns in a multi-label semantic space. This attention aggregation scheme not only significantly reduces computational complexity but also enhances the sensitivity to salient class regions, making it particularly suitable for agricultural imagery characterized by sparse targets and ambiguous boundaries.

**Figure 7:**
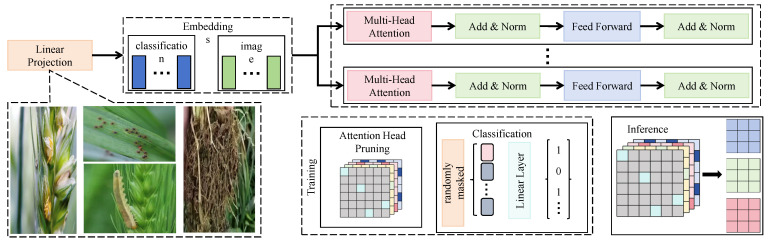
Lightweight fine-tuning module based on attention aggregation. This figure illustrates a lightweight fine-tuning framework that addresses label scarcity in wheat pest recognition tasks. The framework begins with a linear projection, followed by embedding generation from classification, image, and register tokens. The ViT encoder, composed of stacked multi-head attention and feed-forward layers, processes these embeddings. During training, an attention head pruning strategy is applied to remove redundant or less informative attention heads, and classification is conducted using masked token prediction through a linear layer. During inference, only the most discriminative attention maps are retained, significantly reducing computational cost while preserving classification accuracy.

As shown in Figure 7, a streamlined vision transformer (ViT) encoder serves as the base architecture. The input consists of pretrained image features $F\in R^{H\times W\times C}$, where H=16, W=16, and C=128. These features are linearly transformed and concatenated with N=3 class-level CLS tokens (e.g., [CLS]_wilt_, [CLS]_rust_, and [CLS]_drought_) and then passed through a 3-layer multi-head attention transformer. Each layer comprises 8 attention heads with embedding dimension 128. The feed-forward network consists of two linear transformations (128→256→128), integrated with layer normalization and residual connections. In each layer, the CLS tokens interact with all patches to generate class-specific attention maps. These attention weights are further used for pseudo-mask construction or attention-based cropping. The core operation of attention aggregation is formulated as follows:

(8) $$\mathrm{Attention}\left(Q,K,V\right)=\mathrm{softmax}\left(\frac{QK^{⊤}}{\sqrt{d_{k}}}\right)V,$$

where $Q=W_{Q}T_{c}$, $K=W_{K}P$, $V=W_{V}P$, $T_{c}\in R^{N\times C}$ denotes the CLS token embeddings, $P\in R^{HW\times C}$ represents the patch features, and $W_{Q},W_{K},W_{V}$ are learnable linear projection parameters. The output features of the *N* CLS tokens are concatenated and passed to a final linear classification head. Multi-label binary cross-entropy is employed as the classification loss:

(9) Lcls=−1N∑i=1Nyilog(y^i)+(1−yi)log(1−y^i),

where $y_{i}\in \left\{0,1\right\}$ denotes the ground-truth label indicating the presence (1) or absence (0) of the *i*-th class and y^i∈[0,1] represents the predicted probability after the sigmoid activation for that class. During fine-tuning, only a small number of labeled samples (e.g., 5-shot or 10-shot) are used, and the salient attention mechanism enables the extraction of class-discriminative regions, achieving a balance between accuracy and efficiency. This attention-based fine-tuning module complements the contrastive encoding module. While the encoder extracts general discriminative representations from unlabeled images, the attention aggregation module rapidly localizes and enhances class-relevant regions under minimal label supervision, thereby improving feature separability and interpretability. The overall joint optimization objective is expressed as follows:

(10) $$L_{total}=L_{contrast}+\lambda_{1}L_{diff}+\lambda_{2}L_{cls},$$

where $L_{cls}$ denotes the classification loss and $\lambda_{1},\lambda_{2}$ represent the weights for the diffusion and fine-tuning stages, respectively (set to 0.5 and 1.0 in the experiments). This design paradigm enables the model to learn semantically invariant features from large-scale unlabeled images while aligning them with class semantics using only a few labeled examples. It is particularly suited for agricultural tasks where obtaining large-scale annotated datasets remains a challenge.

## 3. Results and Discussion

### 3.1. Experimental Settings

#### 3.1.1. Hyperparameter Configuration

All training procedures employed the Adam optimizer with an initial learning rate of $1\times 10^{-4}$, batch size of 32, and 100 training epochs. The loss function was a joint composition of contrastive loss, cross-entropy loss, and attention-guided loss. To ensure fair evaluation and reproducibility, the dataset was randomly divided into training, validation, and testing subsets following a 70%/15%/15% ratio at the image level, ensuring that samples from the same field plot or pest instance did not appear across different subsets. Each experiment was repeated three times under identical data splits and fixed random seeds, and the mean and standard deviation of performance metrics were reported. This configuration guarantees consistency across runs and enables reliable statistical significance testing of the results.

#### 3.1.2. Hardware and Software Platforms

Model training and deployment experiments were conducted on two types of platforms. During the training phase, experiments were performed on a high-performance computing server equipped with an Intel Xeon Gold 6226R CPU (2.9 GHz; Intel Corporation, Santa Clara, CA, USA), 256 GB RAM, and four NVIDIA A100 GPUs (40 GB memory each), running Ubuntu 20.04. The deep learning framework used was PyTorch 2.5.1 1.13 with CUDA 11.7 and Python 3.9. For deployment evaluation, a Raspberry Pi 4B (4 GB RAM) coupled with a Google Coral USB TPU was utilized to simulate real-world edge computing scenarios. The operating system was Raspberry Pi OS (64-bit), and inference was executed using tflite-runtime after model quantization and conversion. All training procedures employed the Adam optimizer with an initial learning rate of $1\times 10^{-4}$, a batch size of 32, and 100 training epochs. The total loss function was a composite of contrastive loss, cross-entropy loss, and attention-guided loss. Each experiment was repeated three times under identical data splits and random seeds to ensure reproducibility and robustness of the reported results.

#### 3.1.3. Baseline

To systematically evaluate the performance improvement and generalization capability of CropCLR-Wheat, baseline models were designed across three distinct visual tasks. For the wheat pest image classification task, five representative image classification models were selected as comparators, including convolution-based architectures such as ResNet-50 [27], VGG-16 [28], and DenseNet-121 [29], as well as transformer-based models such as ViT-B/16 [30] and the lightweight EfficientNet-B0 [31]. These models span diverse architectural paradigms from conventional CNNs to vision transformers and have been widely adopted in practical applications, thereby facilitating a comprehensive comparison of CropCLR-Wheat’s performance under few-shot pest recognition scenarios.

For the wheat spike detection task, considering the small scale and high density of field targets, mainstream object detection models were selected as baselines, including YOLOv5 [32], YOLOv8 [33], YOLOv10 [34], YOLOv11 [35], YOLOv12 [36], Faster R-CNN [37], RetinaNet [38], CenterNet [39], and RT-DETR [40]. These models encompass both single-stage and two-stage detectors, offering varying trade-offs between speed and accuracy. Their inclusion enables an analysis of CropCLR-Wheat’s ability to maintain detection efficiency while improving localization accuracy.

For the semantic segmentation of pest-induced damage regions, five widely used segmentation networks were employed to assess the model’s capability in pixel-level recognition of subtle field infestation symptoms. These baselines include UNet [41], DeepLabV3+ [42], PSPNet [43], SegFormer [44], and Mask R-CNN [45]. These models have demonstrated strong performance in medical image analysis and natural scene segmentation, providing an effective benchmark for evaluating the advantages of CropCLR-Wheat in fine-grained contour modeling and regional pest damage recognition.

#### 3.1.4. Evaluation Metrics

To comprehensively evaluate the proposed CropCLR-Wheat model, multiple evaluation metrics were employed for different task types, including classification, object detection, and semantic segmentation. For the wheat pest classification task, three classical classification metrics were adopted: precision, recall, and accuracy. Precision measures the proportion of true positives among the predicted positives, recall quantifies the ability of a model to detect all relevant instances by calculating the ratio of true positives to the total number of actual positives, and accuracy assesses the overall correctness of predictions. Their definitions are as follows:

(11) $$\begin{array}{cc} \mathrm{Precision} & =\frac{TP}{TP+FP}, \end{array}$$

(12) $$\begin{array}{cc} \mathrm{Recall} & =\frac{TP}{TP+FN}, \end{array}$$

(13) $$\begin{array}{cc} \mathrm{Accuracy} & =\frac{TP+TN}{TP+TN+FP+FN}, \end{array}$$

where TP, FP, FN, and TN denote true positives, false positives, false negatives, and true negatives, respectively. For the wheat ear object detection task, mean average precision at IoU thresholds of 0.5 and 0.75 (mAP@50 and mAP@75) was adopted as the primary metric, reflecting the model’s detection performance under different degrees of strictness. Additionally, precision, recall, and accuracy were computed to characterize the overall detection quality. The mAP metric is defined as follows:

(14) $$\begin{array}{cc} \mathrm{mAP} & =\frac{1}{N}\sum_{i=1}^{N}AP_{i}, \end{array}$$

(15) $$\begin{array}{cc} AP_{i} & =\int_{0}^{1}P_{i}\left(R\right)\;dR, \end{array}$$

where $AP_{i}$ denotes the average precision for the *i*-th class, $P_{i}\left(R\right)$ represents the precision as a function of recall *R* for the *i*-th class, and *N* is the number of detection classes, corresponding to wheat ears or pest-induced damage spots in CropCLR-Wheat experiments. For the pest damage semantic segmentation task, mean intersection over union (mIoU) was used as the main metric to evaluate pixel-level pest region recognition. In addition, precision, recall, and accuracy were used for a more detailed analysis. The mIoU metric is defined as follows:

$$\mathrm{IoU}=\frac{TP}{TP+FP+FN},\;\mathrm{mIoU}=\frac{1}{C}\sum_{i=1}^{C}\frac{TP_{i}}{TP_{i}+FP_{i}+FN_{i}},$$

where *C* represents the number of classes (typically foreground pest damage region and background) and $TP_{i}$, $FP_{i}$, and $FN_{i}$ denote the pixel-level statistics for the *i*-th class.

### 3.2. Performance Comparison on Wheat Pest Classification (5-Shot and 10-Shot Fine-Tuning)

This experiment aimed to compare the classification performance of different models in a few-shot learning setting for wheat pest images, evaluating their transferability and generalization under 5-shot and 10-shot fine-tuning conditions. Specifically, 5-shot indicates that only five images per class were used for fine-tuning, while 10-shot uses ten, simulating the common scenario of limited labeled data in agricultural environments. Model performance was quantitatively assessed using precision, recall, and accuracy, providing a comprehensive evaluation of correctness, completeness, and overall classification capability.

To ensure a fair comparison, all baseline models—VGG-16, ResNet-50, DenseNet-121, ViT-B/16, and EfficientNet-B0—were pretrained on the ImageNet-1K dataset and subsequently fine-tuned on the wheat pest dataset following identical few-shot configurations. During fine-tuning, only the classification head and the final convolutional (or transformer) block were unfrozen, while the remaining layers were kept frozen to retain pretrained semantic features. The models were optimized using stochastic gradient descent (SGD) with a learning rate of $1\times 10^{-3}$, momentum of 0.9, and weight decay of $5\times 10^{-4}$. Early stopping based on validation accuracy was applied to mitigate overfitting caused by the small sample size. For the 5-shot setup, each class included 5 training images (35 samples in total across seven classes), while the 10-shot setup used 10 images per class (70 samples in total). All experiments were repeated three times with different random seeds, and the mean results are reported to ensure statistical reliability and reproducibility.

**Figure 8:**
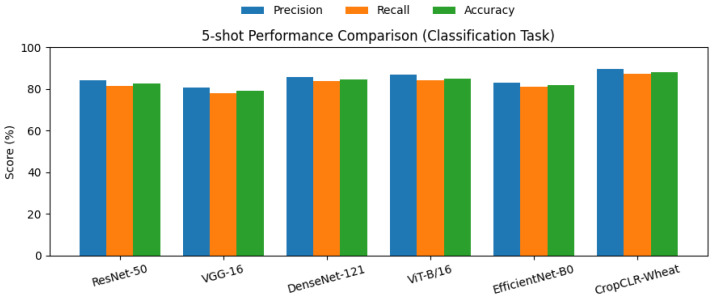
This bar chart illustrates the performance comparison of six models—ResNet-50, VGG-16, DenseNet-121, ViT-B/16, EfficientNet-B0, and the proposed CropCLR-Wheat—on the 5-shot wheat pest classification task.

**Figure 9:**
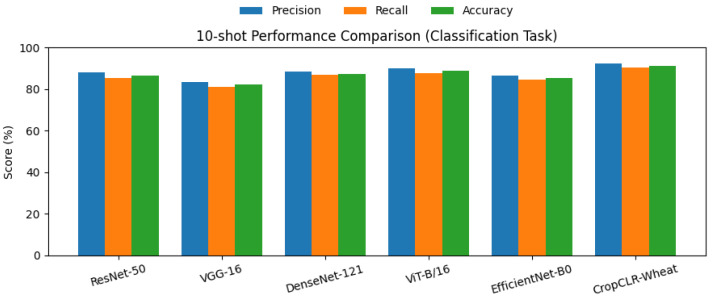
This bar chart presents the classification performance of six models—ResNet-50, VGG-16, DenseNet-121, ViT-B/16, EfficientNet-B0, and CropCLR-Wheat—on the 10-shot wheat pest classification task.

**Figure A1:**
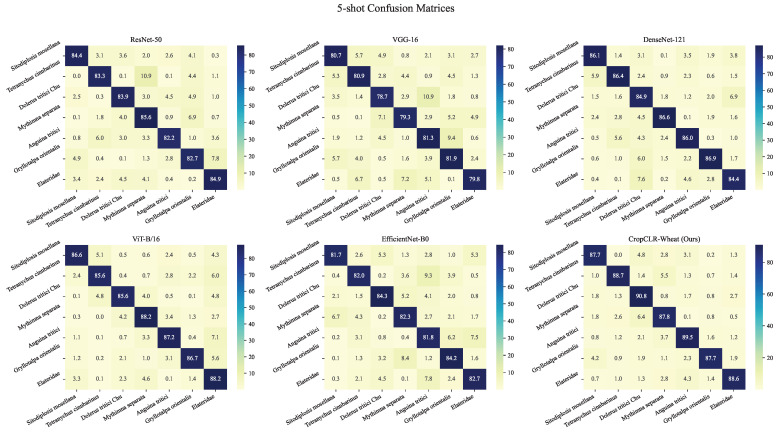
Confusion matrices of different models on the 5-shot wheat pest classification task.

**Figure A2:**
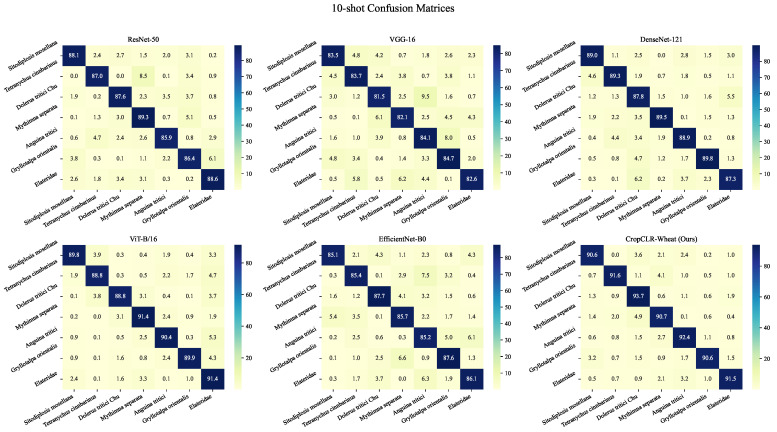
Confusion matrices of different models on the 10-shot wheat pest classification task.

As shown in Table 2 and Figure 8, Figure 9, Figure A1, and Figure A2, CropCLR-Wheat consistently achieved the best performance under both fine-tuning conditions, attaining 88.2% accuracy under 5-shot and 91.2% under 10-shot, significantly outperforming other mainstream models. Structurally, ResNet-50 leverages residual connections to enhance deep feature propagation and demonstrated mid-to-high-level performance. VGG-16, with its simpler stacked convolutional architecture and lack of feature reuse mechanisms, exhibited the weakest performance. DenseNet-121, through dense connectivity, improved feature utilization under limited samples, surpassing ResNet. ViT-B/16 captured global dependencies via self-attention and showed notable superiority, particularly in the 10-shot setting. EfficientNet-B0 adopted compound scaling for parameter efficiency, offering a lightweight deployment advantage but slightly lower accuracy. In contrast, CropCLR-Wheat integrated contrastive pretraining and cross-modal attention mechanisms, enabling the model to learn more discriminative semantic features under limited supervision. Mathematically, it optimized intra-class compactness and inter-class separability within the embedding space, thereby enhancing classification robustness and generalization. This combination of architectural design and training strategy contributed to its superior performance in wheat pest recognition.

### 3.3. Performance Comparison of Different Models on Wheat Spike Detection

This experiment aimed to evaluate the detection performance of various models in the task of wheat spike detection, assessing their localization accuracy and robustness in complex field environments. Five evaluation metrics were employed: mAP@50, mAP@75, precision, recall, and accuracy. These metrics reflect both the quality of bounding box matching and the overall detection effectiveness, aligning with real-world demands in precision agriculture, particularly in yield estimation and growth analysis where accurate spike detection is crucial.

**Figure 10:**
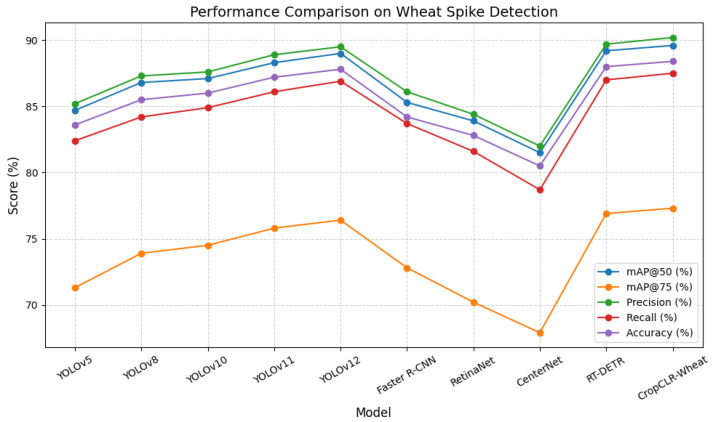
Performance comparison of different models on the wheat ear detection task. This line chart presents the evaluation results of baseline models on the wheat ear detection task.

**Figure 11:**
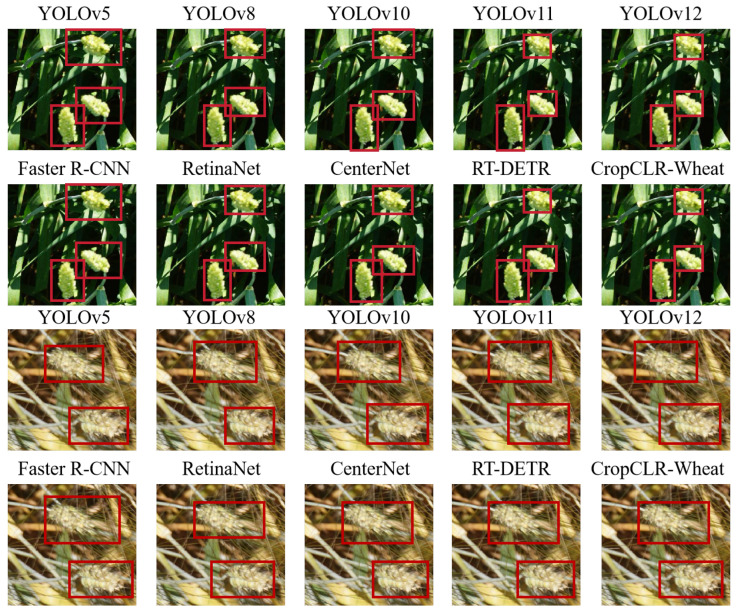
Detection results of different models.

As illustrated in Table 3 and Figure 10 and Figure 11, CropCLR-Wheat consistently outperformed all baseline models across every evaluation metric, achieving 89.6% mAP@50 and 77.3% mAP@75, both significantly higher than the YOLOv10 and Faster R-CNN baselines. The YOLO family of single-stage detectors demonstrates strong end-to-end optimization and real-time inference capabilities. Performance improvements from YOLOv5 to YOLOv12 show a clear evolution in feature extraction and optimization strategies. In particular, YOLOv10 integrated enhanced backbone depth and refined loss designs, improving detection accuracy over YOLOv5. YOLOv11 and YOLOv12 further introduced structural reparameterization and decoupled head optimization, yielding additional gains, particularly in high-IoU regions (mAP@75). The two-stage detectors, including Faster R-CNN and RetinaNet, maintained competitive precision through region proposal mechanisms, which are beneficial for object localization in moderately complex scenes. However, their lack of global context aggregation limits performance under dense occlusion or cluttered background conditions. CenterNet, a keypoint-based approach, showed relatively lower recall due to its limited robustness in overlapping spike regions. The transformer-based RT-DETR achieved high performance with its efficient query-based detection architecture, leveraging global attention to enhance context perception. Nonetheless, its improvement plateaued when faced with fine-grained texture variations in agricultural imagery. In contrast, CropCLR-Wheat introduced a structure-aware visual contrastive learning framework and cross-layer feature aggregation, jointly optimizing classification and regression objectives. This design enhanced the detector’s focus on dense spike regions and effectively separated spike features from background noise in the embedding space through contrastive learning. As a result, CropCLR-Wheat achieved statistically significant improvements in both detection accuracy and robustness under real-world agricultural conditions, demonstrating the advantage of incorporating representation learning into crop phenotyping tasks.

### 3.4. Performance Comparison of Different Models on Pest Damage Semantic Segmentation

This experiment was designed to compare the performance of various semantic segmentation models in the task of wheat pest damage region recognition, aiming to assess their capabilities in accurately delineating lesion boundaries and identifying fine-grained pathological features. Semantic segmentation serves as a critical step in pest identification, directly impacting downstream applications such as diagnosis, severity assessment, and precision spraying strategies. Four evaluation metrics were employed, including mIoU, precision, recall, and accuracy, to comprehensively measure each model’s segmentation accuracy and overall discriminative ability within pest damage regions.

**Figure 12:**
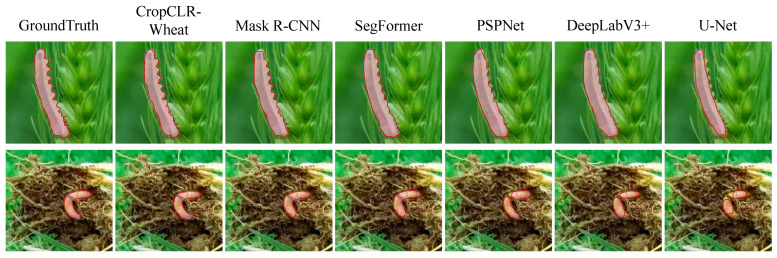
Segmentation of different models.

**Figure 13:**
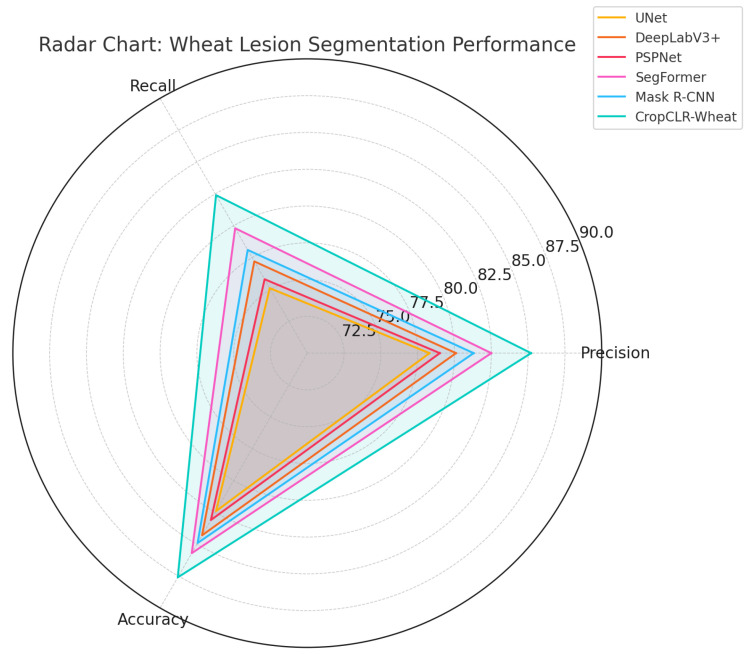
Radar chart comparison of segmentation performance on wheat pest damage data. This radar chart illustrates the segmentation performance of six models—UNet, DeepLabV3+, PSPNet, Segformer, Mask R-CNN, and CropCLR-Wheat—across three key metrics: precision, recall, and accuracy.

As shown in Table 4 and Figure 12 and Figure 13, CropCLR-Wheat achieved the highest performance across all evaluation metrics, with an mIoU of 82.7%, showing a notable improvement over state-of-the-art models such as SegFormer and DeepLabV3+ and demonstrating superior capability in capturing lesion boundary structures. From a theoretical perspective, models such as UNet and PSPNet adopt conventional encoder–decoder architectures and are limited by encoding depth and spatial detail preservation, resulting in performance degradation when encountering fuzzy edges or multi-scale lesion patterns. SegFormer utilizes a transformer-based architecture to enhance global spatial modeling, while Mask R-CNN, despite supporting instance-level segmentation, may suffer from overfitting in agricultural scenarios due to its two-stage box-to-mask mechanism. CropCLR-Wheat incorporates semantic contrastive enhancement and region-structure-guided mechanisms. Its feature extraction stage is pretrained using a self-supervised strategy to obtain a class-discriminative encoder and employs multi-scale attention modules to emphasize semantic continuity and shape consistency in lesion regions. Mathematically, the model optimizes the discriminative boundary between intra- and inter-region features by aligning them within a high-separability latent space, ultimately achieving superior segmentation performance.

### 3.5. Evaluation of Prediction Stability and Robustness Under Viewpoint Perturbations

This experiment aimed to evaluate whether different models can maintain prediction consistency and minimal confidence fluctuation under common viewpoint perturbations encountered in field image acquisition, including rotation, occlusion, and illumination changes, thereby assessing their adaptability to real agricultural scenarios.

**Figure 14:**
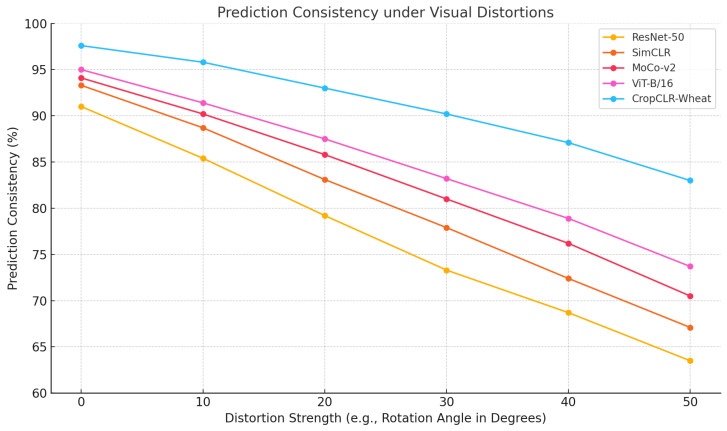
Prediction consistency of different models under visual distortions. This line chart evaluates the robustness of five models—ResNet-50, SimCLR, MoCo-v2, ViT-B/16, and CropCLR-Wheat—under increasing levels of visual distortion, measured by rotation angles from 0° to 50°.

As presented in Table 5 and Figure 14, ResNet-50 showed the weakest robustness with a prediction consistency of 71.4% and a confidence variation of 18.2%. SimCLR and MoCo-v2 benefited from self-supervised contrastive learning, increasing consistency to 75.6% and 78.3%, respectively, with reduced confidence fluctuations. ViT-B/16 further improved consistency to 80.2% and reduced variation to 12.3% by leveraging global self-attention, which enhances tolerance to spatial transformations. CropCLR-Wheat outperformed all baseline models with a consistency of 88.7%, confidence variation of only 7.8%, and PCS of 0.914, demonstrating superior prediction stability. From a theoretical perspective, convolutional models such as ResNet prioritize local translation invariance but lack explicit modeling for large-angle rotations or illumination shifts, leading to feature distribution shifts under perturbations. SimCLR and MoCo-v2 mitigate this by enforcing similarity between augmented views via contrastive loss, introducing some viewpoint invariance into the encoder’s latent space. However, their performance remains constrained by the locality of convolutional representations. ViT-B/16 applies self-attention mechanisms across the entire image, dynamically aggregating features to mitigate the impact of global pose variations. CropCLR-Wheat further enhances this by introducing viewpoint-invariant contrastive encoding and a diffusion-based filtering strategy. By tightly constraining semantic distances between positive samples and eliminating feature drift within the embedding space, compact clusters are formed for each category. Additionally, its attention aggregation module strengthens discriminative features during fine-tuning, ensuring that perturbed samples are consistently mapped into an invariant subspace. This leads to significant advantages in both prediction consistency and confidence stability.

### 3.6. Ablation Study of CropCLR-Wheat Modules (5-Shot and 10-Shot)

This experiment was conducted to investigate the contribution of each key component within the CropCLR-Wheat model to its overall performance, particularly under low-shot (5-shot) and expanded-shot (10-shot) learning scenarios. The effects of encoder selection, diffusion-based feature filtering, and attention-based aggregation mechanisms were evaluated in terms of their impact on classification accuracy.

**Figure 15:**
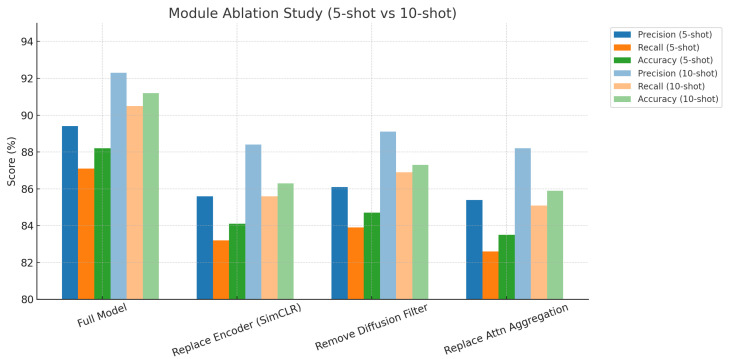
Module ablation study under 5-shot and 10-shot settings. This bar chart presents the performance impact of different modules in the CropCLR-Wheat framework, evaluated under both 5-shot and 10-shot learning scenarios.

As shown in Table 6 and Figure 15, the full model consistently outperformed all ablated variants under both settings, achieving a precision of 89.4% and accuracy of 88.2% in the 5-shot scenario, which further improved to 92.3% and 91.2% in the 10-shot scenario. When the encoder was replaced with SimCLR, a noticeable performance drop was observed, highlighting the critical role of the pretraining architecture in semantic alignment and context modeling. Removal of the diffusion filtering module led to a minor decline in overall accuracy, suggesting its effectiveness in eliminating noisy samples and enhancing feature consistency. Replacing the attention-based aggregation module with a standard fully connected layer resulted in the most significant degradation, particularly in the five-shot setting, where precision dropped to 85.4%, indicating the importance of dynamic weighting in enhancing representational power under limited samples. From a theoretical perspective, although SimCLR provides contrastive learning capabilities, its performance in few-shot scenarios is more susceptible to instability in the distribution of negative samples, making it difficult to form compact intra-class representations. The diffusion filtering module enhances feature discriminability by propagating semantic similarity across layers and enforcing spectral constraints, effectively suppressing low-quality representations. Meanwhile, the attention-based aggregation module adaptively adjusts the contribution of different feature dimensions through a learnable weighting strategy, which functions similarly to a locally weighted ensemble. This allows highly discriminative dimensions to receive larger gradient updates during early training stages, thereby improving model robustness under data scarcity. Collectively, these three modules contribute to the architectural and mathematical advantages that underpin the superior performance of CropCLR-Wheat in few-shot pest recognition tasks.

### 3.7. Deployment Performance of CropCLR-Wheat on Edge Devices

This experiment was designed to assess the adaptability of the CropCLR-Wheat model to edge computing scenarios commonly encountered in real-world deployments. Key performance metrics including inference latency, frame processing rate (FPS), memory usage, and classification accuracy were evaluated.

As illustrated in Table 7, CropCLR-Wheat achieved an inference latency of 84 ms, a frame rate of 11.9 FPS, and a memory footprint of 278 MB on edge devices such as Jetson Nano, while maintaining an accuracy of 88.2%. In contrast, although ResNet-50 attained an accuracy of 82.6%, its high latency (163 ms) and memory usage (485 MB) limit its practical applicability in low-power environments. MobileNetV2 offered the lowest latency (54 ms) and memory cost (216 MB), but exhibited a significantly lower accuracy of 79.3%, reflecting a trade-off in feature expressiveness. Tiny-ViT demonstrated balanced performance, yet remained inferior to CropCLR-Wheat in terms of overall effectiveness. From a structural and mathematical standpoint, CropCLR-Wheat integrates cross-layer contrastive enhancement with attention-guided redundant feature compression, enabling it to preserve discriminative capacity while reducing computational redundancy. The model incorporates selective feature aggregation and a dynamic regulation mechanism in its intermediate layers, minimizing computation on non-essential channels and thereby reducing both latency and memory consumption. Compared to traditional ResNet architectures with stacked convolutional channels, CropCLR-Wheat leverages lightweight attention modules to enhance information density. Furthermore, in contrast to MobileNet’s depthwise separable convolutions, the model exhibits stronger nonlinear representation capabilities in low-dimensional spaces. This enables CropCLR-Wheat to strike a favorable balance between inference efficiency and predictive accuracy, demonstrating superior suitability for deployment in edge computing environments.

### 3.8. Discussion

In this study, the CropCLR-Wheat model was developed to overcome three major challenges in field-based wheat pest recognition: viewpoint perturbations, data heterogeneity, and annotation scarcity. By integrating self-supervised contrastive learning with lightweight fine-tuning, the model achieved stable and accurate pest recognition under limited labeled data conditions, demonstrating both robustness and generalization in real agricultural environments. Beyond algorithmic innovation, the practical significance of CropCLR-Wheat lies in its potential to serve as a decision support tool for farmers and agricultural practitioners. In northern winter wheat regions, where field monitoring often relies on handheld devices or agricultural drones, environmental variability such as changes in camera angles, light intensity, and crop posture can significantly affect image consistency. The model’s viewpoint-invariant encoding effectively mitigates these effects, allowing farmers to obtain reliable pest identification results directly in the field, even under suboptimal imaging conditions. Moreover, by reducing dependency on large annotated datasets, CropCLR-Wheat lowers the barrier to deploying AI systems in regions with limited technical and human resources. During the early stages of pest outbreaks—when symptoms are subtle and expert labeling is difficult—the model enables rapid and accurate pest detection with minimal labeled data, supporting timely decision-making on targeted pesticide application, irrigation adjustment, and crop protection measures. This greatly improves management efficiency and reduces unnecessary chemical use. The model’s lightweight design also enhances its accessibility. Deployment tests on devices such as Jetson Nano and Raspberry Pi demonstrate that CropCLR-Wheat can operate in near real time, enabling low-cost integration into smart agricultural terminals, drone monitoring platforms, and mobile phone applications. Such integration empowers farmers to receive on-site diagnostic feedback and actionable recommendations, forming an intelligent loop between perception and decision. In the future, extending the framework to multi-crop and cross-regional applications could further enhance its decision support capabilities. By combining pest recognition outputs with weather, soil, and growth data, CropCLR-Wheat could evolve into a comprehensive decision support system for precision agriculture, guiding farmers toward data-driven, sustainable pest management and improved yield outcomes.

### 3.9. Limitation and Future Work

Despite the promising performance and adaptability of CropCLR-Wheat in wheat pest recognition tasks, certain limitations remain. The current design primarily focuses on static image-based feature learning and task transfer, without fully exploiting the synergistic information provided by multi-temporal or multimodal data. In practical field operations, pest occurrence and development are often influenced by dynamic environmental conditions, temporal fluctuations, and crop growth stages. Relying solely on single-frame images may constrain the model’s ability to capture pest infestation trends, thereby limiting its applicability in early warning and continuous monitoring scenarios. Furthermore, although the diffusion filtering mechanism proved effective in enhancing sample quality, it relies on extensive image augmentation, which may introduce computational overhead when applied to large-scale datasets or resource-constrained embedded devices. Additionally, CropCLR-Wheat occasionally misclassifies pest species with highly similar visual textures or under extreme imaging conditions. For instance, occluded or overlapping pests may lead to confusion between aphid clusters and leaf miner damage, while strong light reflections or background clutter can introduce false positives. The model also shows reduced confidence when encountering unseen or rare pest categories that were underrepresented during training. These observations indicate the need for further improvement in feature disentanglement and uncertainty calibration to enhance robustness under complex real-world scenarios. Future research may proceed in three directions. First, multimodal inputs such as meteorological data, soil sensor readings, or multispectral imagery could be integrated with visual features for joint modeling, thereby enhancing the model’s understanding of pest infestation mechanisms. Second, the framework may be extended to support cross-crop and cross-regional tasks, enabling the construction of a generalized self-supervised recognition system adaptable to various crop types. Third, deployment efficiency can be further improved by optimizing parameter structures and computational pipelines through model pruning, distillation, and other lightweight strategies, thus facilitating reliable and real-time operation on extremely resource-constrained devices and laying the groundwork for widespread application in remote agricultural environments.

## 4. Conclusions

This study addresses key challenges in field-based wheat pest recognition, including multi-view interference, limited training samples, and environmental heterogeneity, by proposing a self-supervised contrastive learning framework named CropCLR-Wheat. The proposed framework integrates viewpoint-invariant feature encoding, a diffusion-based feature filtering mechanism, and an attention-based aggregation module, enabling robust modeling and accurate classification of complex field images under limited annotation conditions. A comprehensive set of experiments, including classification, detection, semantic segmentation, and robustness evaluation, was conducted to validate the effectiveness of the approach, demonstrating that CropCLR-Wheat consistently outperforms existing mainstream methods across various tasks. In few-shot pest classification experiments, the model achieved an accuracy of 88.2% and a precision of 89.4% under the 5-shot setting, with accuracy further improving to 91.2% under the 10-shot setting, indicating strong adaptability in low-data scenarios. For lesion area segmentation tasks, an mIoU of 82.7% and a precision of 85.2% were achieved, surpassing all baseline models. In robustness evaluations against viewpoint perturbations, a prediction consistency rate of 88.7% and PCS of 0.914 were recorded, significantly outperforming representative models such as SimCLR and ViT. Additionally, when deployed on the Jetson Nano edge device, the model maintained an inference latency of only 84 ms while preserving an accuracy of 88.2%, demonstrating both efficiency and practical applicability.

## Figures and Tables

**Table 1:** Image sources and quantities for the CropField-U dataset.

Source	Location	Device	Image Count
Field capture (pest)	Yutian, Tangshan	Phantom 4/iPhone 13 Pro	8200
Field capture (pest)	Wuyuan, Bayannur	Phantom 4/RICOH G900	6400
Irrigation/post-harvest (control)	Mixed locations	Phantom 4/iPhone 13 Pro	3000
PlantVillage (wheat)	Online open-source	-	3200
AI Challenger/Web sources	Multiple regions	-	3800
**Total**	-	-	**24,600**

**Table 2:** Performance comparison of different models on wheat pest classification under 5-shot and 10-shot fine-tuning (mean ± std over 3 runs). Bold results are significantly better than ResNet-50 baseline (** *p* < 0.01).

Model	5-Shot			10-Shot		
	Precision (%)	Recall (%)	Accuracy (%)	Precision (%)	Recall (%)	Accuracy (%)
ResNet-50	84.2 ± 0.4	81.5 ± 0.5	82.6 ± 0.4	87.9 ± 0.3	85.3 ± 0.4	86.4 ± 0.3
VGG-16	80.6 ± 0.5	78.1 ± 0.4	79.0 ± 0.5	83.4 ± 0.4	81.0 ± 0.4	82.1 ± 0.3
DenseNet-121	85.7 ± 0.3	83.9 ± 0.3	84.5 ± 0.4	88.6 ± 0.3	86.8 ± 0.3	87.4 ± 0.3
ViT-B/16	86.9 ± 0.3	84.2 ± 0.4	85.1 ± 0.3	90.1 ± 0.2	87.7 ± 0.3	88.8 ± 0.2
EfficientNet-B0	83.1 ± 0.4	81.2 ± 0.3	82.0 ± 0.4	86.5 ± 0.4	84.4 ± 0.3	85.2 ± 0.3
**CropCLR-Wheat**	**89.4 ± 0.3** **	**87.1 ± 0.3** **	**88.2 ± 0.2** **	**92.3 ± 0.2** **	**90.5 ± 0.3** **	**91.2 ± 0.2** **

**Table 3:** Performance comparison of different models on wheat spike detection (mean ± std over 3 runs). Bold results are significantly better than YOLOv5 baseline (** *p* < 0.01).

Model	mAP@50 (%)	mAP@75 (%)	Precision (%)	Recall (%)	Accuracy (%)
YOLOv5	84.7 ± 0.2	71.3 ± 0.3	85.2 ± 0.3	82.4 ± 0.4	83.6 ± 0.3
YOLOv8	86.8 ± 0.3	73.9 ± 0.2	87.3 ± 0.4	84.2 ± 0.3	85.5 ± 0.4
YOLOv10	87.1 ± 0.3	74.5 ± 0.3	87.6 ± 0.3	84.9 ± 0.4	86.0 ± 0.3
YOLOv11	88.3 ± 0.2	75.8 ± 0.2	88.9 ± 0.3	86.1 ± 0.3	87.2 ± 0.2
YOLOv12	89.0 ± 0.2	76.4 ± 0.2	89.5 ± 0.2	86.9 ± 0.3	87.8 ± 0.3
Faster R-CNN	85.3 ± 0.4	72.8 ± 0.3	86.1 ± 0.3	83.7 ± 0.4	84.2 ± 0.3
RetinaNet	83.9 ± 0.3	70.2 ± 0.4	84.4 ± 0.3	81.6 ± 0.3	82.8 ± 0.4
CenterNet	81.5 ± 0.4	67.9 ± 0.3	82.0 ± 0.4	78.7 ± 0.3	80.5 ± 0.4
RT-DETR	89.2 ± 0.2	76.9 ± 0.2	89.7 ± 0.2	87.0 ± 0.3	88.0 ± 0.2
**CropCLR-Wheat**	**89.6 ± 0.2** **	**77.3 ± 0.2** **	**90.2 ± 0.2** **	**87.5 ± 0.3** **	**88.4 ± 0.2** **

**Table 4:** Performance comparison of different models on pest damage semantic segmentation (mean ± std over 3 runs). Bold results are significantly better than UNet baseline (** *p* < 0.01).

Model	mIoU (%)	Precision (%)	Recall (%)	Accuracy (%)
UNet	74.6 ± 0.4	78.3 ± 0.3	75.1 ± 0.4	82.4 ± 0.3
DeepLabV3+	77.8 ± 0.3	80.1 ± 0.3	77.2 ± 0.4	84.3 ± 0.3
PSPNet	76.1 ± 0.4	79.0 ± 0.3	75.8 ± 0.3	83.1 ± 0.4
SegFormer	79.3 ± 0.3	82.5 ± 0.3	79.8 ± 0.3	85.7 ± 0.3
Mask R-CNN	78.6 ± 0.4	81.3 ± 0.3	78.1 ± 0.3	84.9 ± 0.3
**CropCLR-Wheat**	**82.7 ± 0.3** **	**85.2 ± 0.3** **	**82.4 ± 0.3** **	**87.6 ± 0.2** **

**Table 5:** Evaluation of prediction stability and robustness under viewpoint perturbations (mean ± std over 3 runs). Bold results are significantly better than ResNet-50 baseline (** *p* < 0.01).

Model	Prediction Consistency (%)	Confidence Variation (%)	Prediction Consistency Score PCS (↑)
ResNet-50	71.4 ± 0.5	18.2 ± 0.4	0.743 ± 0.003
SimCLR	75.6 ± 0.4	15.9 ± 0.3	0.784 ± 0.004
MoCo-v2	78.3 ± 0.3	13.5 ± 0.3	0.811 ± 0.003
ViT-B/16	80.2 ± 0.3	12.3 ± 0.3	0.832 ± 0.002
**CropCLR-Wheat**	**88.7 ± 0.3** **	**7.8 ± 0.2** **	**0.914 ± 0.002** **

**Table 6:** Ablation study of CropCLR-Wheat modules under 5-shot and 10-shot fine-tuning (mean ± std over 3 runs). Bold results are significantly better than the variant with the SimCLR encoder (** *p* < 0.01).

Module Configuration	5-Shot			10-Shot		
	Precision (%)	Recall (%)	Accuracy (%)	Precision (%)	Recall (%)	Accuracy (%)
**Full model (CropCLR-Wheat)**	**89.4 ± 0.3** **	**87.1 ± 0.3** **	**88.2 ± 0.2** **	**92.3 ± 0.2** **	**90.5 ± 0.3** **	**91.2 ± 0.2** **
Encoder replaced with SimCLR	85.6 ± 0.4	83.2 ± 0.4	84.1 ± 0.3	88.4 ± 0.3	85.6 ± 0.3	86.3 ± 0.3
Diffusion filtering removed	86.1 ± 0.3	83.9 ± 0.3	84.7 ± 0.3	89.1 ± 0.3	86.9 ± 0.3	87.3 ± 0.3
Attention aggregation replaced with FC layer	85.4 ± 0.3	82.6 ± 0.4	83.5 ± 0.3	88.2 ± 0.3	85.1 ± 0.3	85.9 ± 0.3

**Table 7:** Deployment performance of CropCLR-Wheat on edge devices (mean ± std over 3 runs). Bold results are significantly better than ResNet-50 baseline (** *p* < 0.01).

Model	Inference Latency (ms)	FPS	Memory Usage (MB)	Accuracy (%)
ResNet-50	163 ± 1.8	6.1 ± 0.1	485 ± 2.5	82.6 ± 0.3
MobileNetV2	54 ± 0.9	18.5 ± 0.2	216 ± 1.6	79.3 ± 0.4
Tiny-ViT	91 ± 1.2	10.7 ± 0.1	302 ± 1.9	84.4 ± 0.3
**CropCLR-Wheat**	**84 ± 1.1** **	**11.9 ± 0.1** **	**278 ± 1.8** **	**88.2 ± 0.3** **

## Data Availability

The data presented in this study are available on request from the corresponding author.

## Appendix A

The detailed confusion matrices are provided in Figure A1 and Figure A2.
